# ASO Author Reflections: The Impact of Minimally Invasive Surgery on the Completion Rate of the Perioperative Chemotherapy Protocol in Gastric Cancer

**DOI:** 10.1245/s10434-023-14023-z

**Published:** 2023-07-31

**Authors:** Andrianos Tsekrekos, David Borg, Victor Johansson, Magnus Nilsson, Fredrik Klevebro, Lars Lundell, Maria Gustafsson-Liljefors, Ioannis Rouvelas

**Affiliations:** 1 Department of Upper Abdominal Diseases, Karolinska University Hospital, Stockholm, Sweden; 2 Division of Surgery and Oncology, Department of Clinical Science, Intervention and Technology (CLINTEC), Karolinska Institutet, Stockholm, Sweden; 3 Oncology Department, Skåne University Hospital, Lund, Sweden; 4 Division of Oncology and Therapeutic Pathology, Department of Clinical Sciences, Lund University, Lund, Sweden; 5 Department of Oncology, Sahlgrenska University Hospital, Gothenburg, Sweden; 6 Department of Surgery, Odense University Hospital, Odense, Denmark

## Past

A multimodal therapeutic strategy that combines radical surgery with perioperative chemotherapy is the standard of care for the curative treatment of locally advanced gastric cancer (AGC) in Europe. However, intolerance for the postoperative chemotherapy is a frequent clinical problem. The randomized trials that provided the evidence on the survival advantage of this approach also demonstrated that approximately 50% of the operated patients were not able to receive the designated adjuvant chemotherapy.^1,2^ The same became evident in the more recent FLOT4-AIO trial.^3^ This is mainly due to operative morbidity or compromised general condition and nutritional status, which are common after gastrectomy. During the past 2 decades, laparoscopic gastrectomy (LG) for AGC has been implemented worldwide. Several trials have compared LG to open gastrectomy (OG) with regard to short-term outcomes (operative morbidity and mortality, oncological surgical quality) and long-term survival, showing that LG yields non-inferior and in some aspects superior results, e.g. a shorter convalescence period. Nevertheless, whether the advantage of a more favorable course after LG translates into better compliance with the ensuing chemotherapy has not been fully investigated.

## Present

We analyzed data from the population-based Swedish national register to test the hypothesis that LG has a positive effect on the completion of the perioperative chemotherapy protocol and our results were published recently in the *Annals of Surgical Oncology*.^4^ A total of 247 patients with AGC who had received neoadjuvant chemotherapy and gastrectomy (126 LG and 121 OG) and were intended to proceed with adjuvant chemotherapy were identified in the database. Additional detailed information on the administered chemotherapy was retrieved from medical records. Eighty-six percent of the patients started adjuvant chemotherapy, with no significant difference between the groups (LG 88% vs. OG 84%). Of the patients who started treatment, reduction of chemotherapy occurred in 37% and the proportion was also similar between the two groups (LG 39% vs. OG 35%), as was the time interval from surgery to start of treatment. There was no association between the surgical approach and the probability of starting treatment or the need for chemotherapy reduction in the multivariable analysis. Conversely, severe, postoperative complications had a significant, negative impact on both outcomes.

## Future

Studies suggest that the adjuvant component of the perioperative chemotherapy contributes substantially to the overall positive effect on survival.^5^ Our study did not show any benefit of the laparoscopic approach in terms of rate of initiation, need for modification, and time to start of the adjuvant treatment. In contrast, we found that severe postoperative complications were strongly associated with omission of adjuvant chemotherapy and an increased likelihood of chemotherapy reduction, which occurred in 44% of the enrolled patients. Thus, it became clear that postoperative morbidity is a crucial determinant of the ability to resume the oncological treatment. Considering that morbidity rates as high as 40% are reported in Western series, further exploration of multidisciplinary interventions in perioperative care with focus on minimizing postoperative complications is warranted.
